# Infections or Vaccines Associated with Finkelstein-Seidlmayer Vasculitis: Systematic Review

**DOI:** 10.1007/s12016-022-08940-2

**Published:** 2022-05-12

**Authors:** Gabriel Bronz, Céline Betti, Pietro O. Rinoldi, Lisa Kottanattu, Mario G. Bianchetti, Danilo Consolascio, Marcel M. Bergmann, Gregorio P. Milani, Benedetta Terziroli Beretta Piccoli, Sebastiano A. G. Lava

**Affiliations:** 1 Pediatric Institute of Southern Switzerland, Ente Ospedaliero Cantonale, Bellinzona, Switzerland; 2 Faculty of Biomedical Sciences, Università Della Svizzera Italiana, Lugano, Switzerland; 3 Family Medicine, Faculty of Biomedical Sciences, Università Della Svizzera Italiana, Lugano, Switzerland; 4 Centro Pediatrico del Mendrisiotto, Mendrisio, Switzerland; 5 Pediatric Allergy Unit, Department of Woman, Child and Adolescent, University Hospitals of Geneva, Geneva, Switzerland; 6 Pediatric Unit, Fondazione IRCCS Ca’ Granda Ospedale Maggiore Policlinico, Milan, Italy; 7 Department of Clinical Sciences and Community Health, Università Degli Studi Di Milano, Milan, Italy; 8 Epatocentro Ticino, Lugano, Switzerland; 9 Pediatric Cardiology Unit, Department of Pediatrics, Centre Hospitalier Universitaire Vaudois and University of Lausanne, Lausanne, Switzerland; 10 Heart Failure and Transplantation, Department of Paediatric Cardiology, Great Ormond Street Hospital, London, UK

**Keywords:** Acute hemorrhagic edema of young children, Cockade purpura with edema, Finkelstein-Seidlmayer vasculitis, Infection, Precursor, Small-vessel leukocytoclastic vasculitis, Vaccination

## Abstract

Finkelstein-Seidlmayer vasculitis, also referred to as acute hemorrhagic edema of young children, is a rare small-vessel leukocytoclastic vasculitis. This condition is skin-limited, mainly affects infants up to 2 years of age and spontaneously remits. It has been suggested that an infection or a vaccine precede (by ≤ 14 days) this vasculitis. To better understand the interplay between infections or vaccines and Finkelstein-Seidlmayer vasculitis, we utilized the data contained in the Acute Hemorrhagic Edema BIbliographic Database AHEBID. The database, initiated in 2019, is being regularly updated, encompasses the entire original literature on Finkelstein-Seidlmayer vasculitis published after the original description and is attainable on request. The possible existence of an infectious or a vaccine precursor was addressed in 447 cases. Most cases were preceded by an infection (N = 384; 86%), by a vaccination (N = 20; 4.4%), or both an infection and a vaccination (N = 17; 3.8%). No precursor was reported in the remaining cases (N = 26; 5.8%). Two distinct infections preceded the onset of the vasculitis in 11 of the 381 cases with infection-associated Finkelstein-Seidlmayer vasculitis. The following infectious precursors were reported: upper respiratory tract infection (N = 292); acute gastroenteritis (N = 40); a benign febrile infection (N = 36); lower respiratory tract infection (N = 22); further infections (N = 8). The temporal relationship between the infectious precursor and the onset of the skin eruption was detailed in 336 cases: 54 cases developed before resolution and 282 after resolution of the infection. In conclusion, most cases of Finkelstein-Seidlmayer vasculitis are preceded by an infection. In a minority of cases, this skin vasculitis develops before resolution of the infection. In most cases, however, this vasculitis develops after resolution of the infection. More rarely, this vasculitis is preceded by a vaccination.

**Supplementary Information:**

The online version contains supplementary material available at 10.1007/s12016-022-08940-2.

## Introduction

Finkelstein-Seidlmayer vasculitis, also referred to as acute hemorrhagic edema of young children, cockade purpura with edema, infantile erythema multiforme, or infantile Henoch-Schönlein purpura, is a rare immune-mediated small-vessel leukocytoclastic vasculitis [1–4]. This condition is skin-limited, mainly occurs in infants up to 2 years of age, spontaneously remits in a maximum of 3 weeks and does not tend to recur [1–4]. Affected patients are well-appearing and acutely present with widespread and symmetrically distributed annular or nummular eruptions and edema [1–4]. The annular or nummular eruptions are distributed over the legs, feet, face, arms, ears, trunk, and genitals in that order of frequency. The distribution is identical for edema except for feet, which are very often affected [1–4].

Textbooks and narrative reviews state that Finkelstein-Seidlmayer vasculitis is often preceded by a respiratory tract infection, a gastroenteritis, a urinary tract infection, a benign febrile disease with unknown source, or a vaccine [2, 4]. To better understand the interplay between infections or vaccines and Finkelstein-Seidlmayer vasculitis, we utilized the data contained in the Acute Hemorrhagic Edema BIbliographic Database AHEBID.

## Methods

**Fig. 1:**
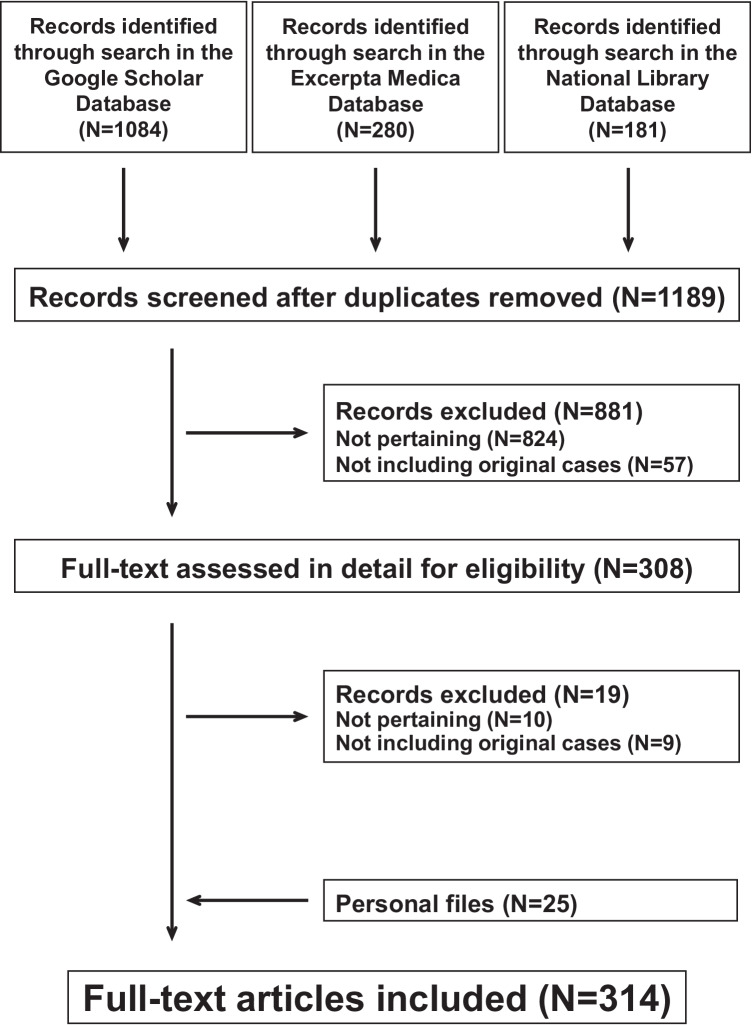
Finkelstein-Seidlmayer vasculitis. Flowchart of the literature search. The preferred reporting items for systematic reviews and meta-analyses were followed

AHEBID was initiated by some of us [3, 5, 6] in 2019, is being regularly updated and encompasses the entire original literature on Finkelstein-Seidlmayer vasculitis published after the original description in 1913 [7]. The database is attainable on request (email: finkelstein-seidlmayer@usi.ch). For this purpose, the bibliography search engines Excerpta Medica, the National Library of Medicine database and Google scholar are screened every second month for “acute hemorrhagic edema”, “cockade purpura and edema”, “Finkelstein-Seidlmayer” and “infantile Henoch-Schönlein purpura” without any language restriction. To increase the rigor of the literature search, we conduct the search in agreement with 2020 version of the Preferred Reporting Items for Systematic Reviews and Meta-Analyses. AHEBID also incorporates secondary references and the literature on Finkelstein-Seidlmayer vasculitis collected by one of us in the early eighties of the last century [8]. As of September 1, 2021, the database (Fig. 1) included 314 (see: supplementary material) original reports (letters, case reports or full-length articles) published after 1969, which addressed 504 (349 boys and 155 girls) individually documented cases: 7 neonates less than 4 weeks of age, 252 infants less than 12 months of age, 181 infants 12 to 23 months of age and 63 children 24 months or more of age (this piece information was not available in one case). For all patients, the diagnosis of hemorrhagic edema made in the original reports was reviewed using three well established clinical criteria: raised annular or nummular eruptions and inflammatory skin edema (mostly non-pitting, tender and sometimes also warmth) in a well-appearing and playful child with stable vital signs [5]. The clinical diagnosis was supported by a skin biopsy [9] disclosing a non-granulomatous neutrophil infiltration into small-vessel walls with karyorrhexis in 248 (50%) cases.

For the present study, we meticulously extracted published cases of Finkelstein-Seidlmayer vasculitis where the existence of an associated infectious or vaccine precursor (by 14 days or less) was discussed [4, 5]. The temporal relationship between infection and onset of Finkelstein-Seidlmayer vasculitis was employed to classify the condition as intra- (skin vasculitis starting before resolution of the infection) or post-infectious (skin disorder starting after resolution of the infection). The term latency was used to denote the time (in days) elapsed from onset of infectious symptoms to onset of skin eruption. The infectious precursors [10–12] were classified as depicted in Table 1.

**Table 1:** Classification of infections utilized for the present analysis

**• Acute upper respiratory tract infection**	
- One or more of the following signs: purulent nasal discharge; cough; red, bulging tympanic membrane with loss of normal landmarks; purulent ear discharge; pharyngo-tonsillar erythema or exudate	
- The term was also employed for patients diagnosed with common cold, pharyngitis, tonsillitis, or otitis media in the original report	
**• Acute lower respiratory tract infection (including pneumonia)**	
- Cough associated with one or more of the following signs: labored breathing; chest indrawing; rales; stridor; wheezing; cyanosis; respiratory rate > 50/min	
- The term was also employed for cases diagnosed with croup, bronchitis, bronchiolitis, or pneumonia in the original report	
**• Acute lower respiratory tract infection (including pneumonia)**	
- Cough associated with one or more of the following signs: labored breathing; chest indrawing; rales; stridor; wheezing; cyanosis; respiratory rate > 50/min	
- The term was also employed for cases diagnosed with croup, bronchitis, bronchiolitis, or pneumonia in the original report	
**• Acute gastroenteritis**	
- Three or more loose stools per day (or a number of bowel movements exceeding the child’s usual number of daily bowel movements by two or more) with or without associated fever and vomiting	
- The term was also employed for cases diagnosed with acute gastroenteritis or acute infectious diarrhea in the original report	
**• Urinary tract infection**	
- Increased body temperature, failure to thrive, poor feeding or acute urinary symptoms associated with urinalysis disclosing a pathological pyuria and a significant positive urine culture (excluding lactobacilli, corynebacteria, and coagulase-negative staphylococci)	
- The term was also employed for cases diagnosed with urinary tract infection or pyelonephritis in the original report	
**• Benign febrile infection without a source**	
- Fever in a previously healthy, otherwise well-appearing child when a complete history and physical examination do not identify a specific source of infection. Occult infections such as urinary tract infection, bacteremia or pneumonia have also been reasonably excluded	
- The term was also employed for cases diagnosed with benign febrile infection, fever without localizing signs or fever without a focus in the original report	
**• Further infections**	
The diagnosis made in the original report was retained	

Categorical variables are shown as counts and were analyzed using the Fisher’s exact test. Numerical variables are presented as medians and interquartile ranges and were compared using the Mann–Whitney U test. Two-sided *P*-values of less than 0.05 were regarded as statistically significant.

## Results

**Fig. 2:**
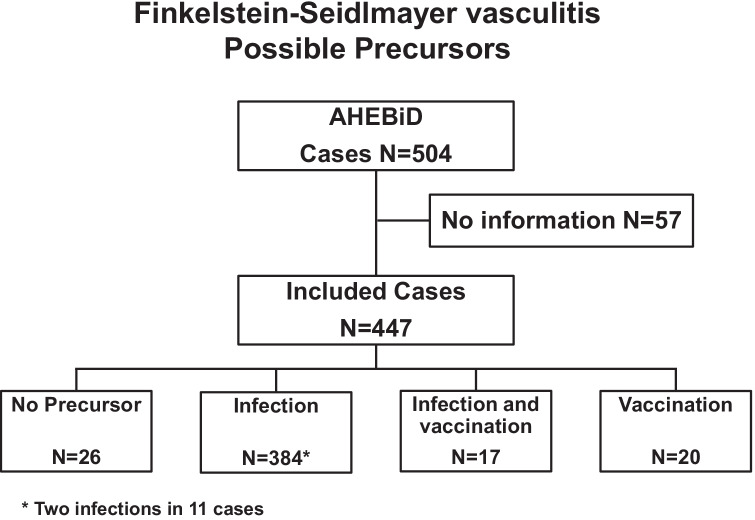
Finkelstein-Seidlmayer vasculitis. Precursors. The term AHEBID denotes the Acute Hemorrhagic Edema BIbliographic Database

The possible existence of an infectious or a vaccine precursor was addressed in 447 cases and not addressed in the remaining 57 cases (Fig. 2). Cases with this piece of information were slightly younger than cases without it (11 [8–18] months of age vs 14 [9–21] months of age, *P* < 0.05). The male-to-female ratio was similar in the two groups (309 boys and 138 girls, respectively, 40 boys and 17 girls). Most cases were preceded by an infection (86%), by a vaccination (4.4%), or both an infection and a vaccination (3.8%). No precursor was reported in the remaining cases (5.8%). Two distinct infections preceded the onset of the skin eruptions in 11 of the 384 cases with infection-associated Finkelstein-Seidlmayer vasculitis.

**Fig. 3:**
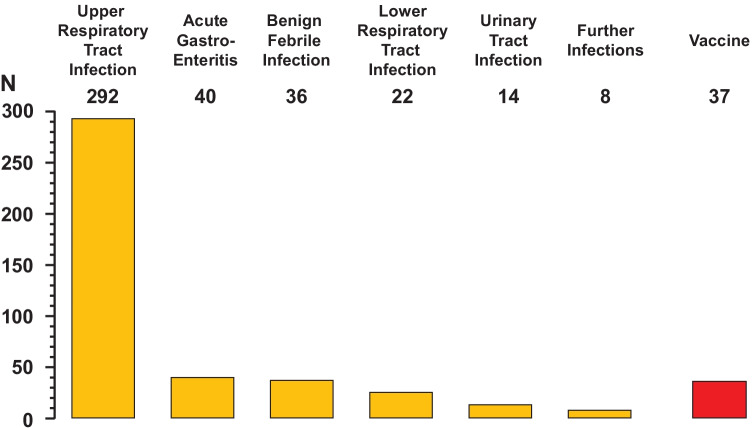
Infectious or vaccine precursors noted in 423 patients with Finkelstein-Seidlmayer vasculitis

The 449 precursors noted in 423 infants are given in Fig. 3. Most cases were preceded by an upper respiratory tract infection (65%), followed by a vaccine (8.2%), a gastroenteritis (8.9%), a benign febrile infection (8.0%) or a lower respiratory infection (4.9%).

Ninety-seven microorganisms possibly causing the infectious precursor were detected in 85 infants: 54 bacteria, 41 viruses, and 2 fungi (Table 2).

**Table 2:** Microorganisms (N = 97) associated with the infectious precursor in 85 patients (65 males and 20 females; 10 [7–14] months of age) with Finkelstein-Seidlmayer vasculitis

	**N**
**Bacteria**	**54**^**a**^
Streptococcus species	20
* Streptococcus pyogenes*	*11*
*Streptococcus pneumoniae*	*5*
*Streptococcus* not otherwise specified	3
*Streptococcus viridans*	1
*Escherichia coli*	12
*Mycoplasma pneumoniae*	6
Staphylococcus species	4
*Staphylococcus aureus*	2
*Staphylococcus* not otherwise specified	2
Campylobacter species	3
*Campylobacter jejuni*	2
*Campylobacter mucosalis*	1
*Proteus mirabilis*	3
*Mycobacterium tuberculosis*	2
Gram-positive bacterium, not otherwise specified	1
Gram-negative bacterium, not otherwise specified	1
*Haemophilus influenzae*	1
*Salmonella* not otherwise specified	1

**Viruses**	**41**^b^
Herpesviridae	9
Human Herpes Virus 3 (Varicella Zoster Virus)	4
Human Herpes Virus 1 (Herpes simplex 1)	2
Human Herpes Virus 4 (Epstein-Barr Virus)	2
Human Herpes Virus 5 (Cytomegalovirus)	1
Adenovirus	5
Rhinovirus	4
Coronaviruses	4
Severe acute respiratory syndrome coronavirus 2	3
Coronavirus NL63	1
Picornaviruses	6
Coxsackievirus not otherwise specified	1
Coxsackievirus B4	1
Coxsackievirus B5	1
Enterovirus not otherwise specified	3
Parvovirus B19	3
Rotavirus	3
Bocavirus	1
Echovirus	1
Hepatitis A virus	1
Human metapneumovirus	1
Parainfluenza virus	1
Paramyxovirus	1

**Fungi**	**2**
*Candida albicans*	2

^a^in 47 patients

^b^in 26 patients

The temporal relationship between the infectious precursor and the onset of the skin eruption was not detailed in 65 cases (47 boys and 18 girls, 11 [8–18] months of age). This parameter was reported in the remaining 336 cases (230 boys and 106 girls, 11 [8–17] months of age). Fifty-three cases (38 boys and 15 girls, 11 [7–14] months of age) were intra- and 283 (191 boys and 92 girls, 11 [8–17] months of age) post-infectious (Table 3). The latency time was significantly lower in the 23 intra-infectious cases (P < 0.001) as compared with the 205 post-infectious cases.

**Table 3:** Characteristics of patients with post- or intra-infectious Finkelstein-Seidlmayer vasculitis. Data are given as median and interquartile range or as frequency

	**Vasculitis**		**P-value**
	**Post-infectious**	**Intra-infectious**	
N	283	53	
Males: females, N	191: 92	38: 15	0.528
Age, months	11 [8–17]	11 [7–14]	0.230
Latency, days	7^a^ [4–14]	2^b^ [1–3]	< 0.001
Precursor			
Upper respiratory infection, N (%)	222 (78%)	31 (58%)	< 0.005
Acute gastroenteritis, N (%)	30 (11%)	4 (7.5%)	0.625
Lower respiratory infection, N (%)	14 (4.9%)	5 (9.4%)	0.204
Urinary tract infection, N (%)	11 (3.9%)	2 (3.8%)	0.999
Benign febrile infection, N (%)	10 (3.5%)	3 (5.7%)	0.445
Further infections, N (%)	5 (1.8%)	9 (17%)	< 0.001

^a^Information available for 205 cases

^b^information available for 23 cases

In 37 cases (24 males and 13 females, 12 [7–18] months of age), the skin eruptions were preceded by one or more active immunizations (Table 4). The temporal relationship between the vaccination and the onset of this vasculitis was not described in 10 cases (8 boys and 2 girls, 17 [8–20] months of age). The latency time was 7 [3–13] days in the remaining 27 cases (16 boys and 11 girls, 12 [7–18] months of age).

**Table 4:** Immunizations (N = 103) potentially implicated in 42 cases of Finkelstein-Seidlmayer vasculitis (29 males and 13 females, 12 [7–18] months of age). The vast majority of the 42 cases were administered a combination vaccine

**Vaccination**	**N**
*Clostridium tetani*	18
*Corynebacterium diphteriae*	18
*Bordetella pertussis*	17
*Haemophilus influenzae* B	10
Poliovirus	9
Measles virus	5
Rubella virus	5
Hepatitis B virus	4
*Streptococcus pneumoniae*	4
Further microbes*	3

^*^Each one case after hepatitis A virus, influenza H1N1 and mumps virus

## Discussion

This systematic literature review documents that most cases of Finkelstein-Seidlmayer vasculitis are preceded by an infection. In a minority of cases, this skin vasculitis develops before resolution of the infection, i.e. intra-infectiously. In the vast majority of cases, however, this vasculitis develops after resolution of the infection, i.e., post-infectiously. More rarely, this vasculitis is preceded by a vaccination or both an infection and a vaccination, indicating that infections by far more frequently trigger immune-mediated diseases than vaccinations.

The reason for the unique distribution of skin lesions with predilection for face and ears remains unclear. The following possible explanations may be offered. In immunoglobulin A vasculitis, the location of skin lesions is notoriously gravity-dependent. Since infants with Finkelstein-Seidlmayer vasculitis spend most of their time lying down, gravity cannot play a role. The main factor that may modulate the distribution of lesions is the blood supply: the proportionally large head and face with a corresponding increase in blood supply in infants predisposes them more to facial lesions. A predilection for the face is observed also in infants with strawberry hemangioma, where the subcutaneous arteries that nourish the hemangioma are mostly found in the head [4]. Finally, it is tempting to assume a role also for skin-resident cells.

Infectious causes have long been suspected for many vasculitis syndromes [13, 14]. In most cases, the vascular injury has been ascribed to immune-mediated mechanisms rather than direct microbial invasion [13, 14]. There is a recognized association between some vasculitides and infections: *Mycobacterium tuberculosis* is likely involved in Takayasu arteritis [15] and a *Burkholderia* in Horton giant cell arteritis [16]; Hepatitis B and C viruses have been associated to polyarteritis nodosa [17]; *Staphylococcus aureus* has been the focus of many studies on granulomatosis with polyangiitis [18]; finally, Hepatitis C virus is a cause of cryoglobulinemia vasculitis [19]. Immunoglobulin A–associated small-vessel leukocytoclastic vasculitis has been associated with bacteria such as group A Streptococci, atypical bacterial pathogens (including Mycoplasma), *Helicobacter pylori*, *Staphylococcus aureus*, *Salmonella enteritidis* or *Campylobacter*, and with viruses such as Parvovirus B19, Varicella-zoster virus, Epstein-Barr virus and especially common respiratory viruses [20]. Very recently, immunoglobulin A vasculitis has also been associated with severe acute respiratory syndrome coronavirus 2 [21].

The precursors noted in Finkelstein-Seidlmayer vasculitis are concurrently similar and different from those observed in immunoglobulin A vasculitis. Both Finkelstein-Seidlmayer vasculitis and immunoglobulin A vasculitis are often preceded either by a vaccination or, more frequently, an infection. However, *Streptococcus pyogenes* and atypical bacterial pathogens including Mycoplasma very often precede immunoglobulin A vasculitis but rather rarely precede Finkelstein-Seidlmayer vasculitis [20, 22, 23]. This difference is likely related to the fact that *Streptococcus pyogenes* [24] and atypical bacterial pathogens [25] rarely affect subjects 4–5 years or less of age. Textbooks and reviews sometimes emphasize an unproven association between Mycoplasma and Finkelstein-Seidlmayer vasculitis. This misunderstanding likely reflects the fact that the clinical hallmarks of this vasculitis resemble erythema multiforme, a condition often caused by Mycoplasma [1–4]. Our data also demonstrate that, like with immunoglobulin A vasculitis, respiratory tract viruses including coronavirus disease 2019 [26] may be associated with Finkelstein-Seidlmayer vasculitis. Vaccination against severe acute respiratory syndrome coronavirus 2 has also been occasionally associated with immunoglobulin A vasculitis [27]. On the contrary, no cases of Finkelstein-Seidlmayer vasculitis have been so far associated with this vaccination, likely because it is currently not recommended in young children. Finally, it is worth of mention that a temporal association between an infection (or a vaccination) and Finkelstein-Seidlmayer vasculitis does not inexorably and in every case imply causality.

Immunoglobulin A vasculitis is obviously an immunoglobulin A dominant vasculitis [2, 4]. Since immunoglobulin A is an early response to a pathogen, the clinical features of this vasculitis mostly occur before resolution of the infectious precursor, i.e., intra-infectiously [2, 4]. Finkelstein-Seidlmayer vasculitis is a non-immunoglobulin A dominant immune-mediated vasculitis. Consequently, the features of this vasculitis mostly occur after resolution of the infection, i.e., post-infectiously.

The results of this systematic review must be viewed with an understanding of the inherent limitations of the analysis process, which integrated information from reports detailing on average one to two cases of Finkelstein-Seidlmayer vasculitis reported over a period of 50 years. For example, the diagnostic strategy was heterogeneous: it was often supported by a skin biopsy study in cases managed by dermatologists but was made clinically in those managed by pediatricians. The obvious strength of this analysis is that it includes the whole literature on this vasculitis over the period 1970–2021.

## Conclusions

In Finkelstein-Seidlmayer vasculitis (like in immunoglobulin A vasculitis), it is typical that different infectious agents may trigger the same pathological damage. On the other hand, a specific microbe may produce more than one vasculitis syndrome. The mechanisms underlying the occurrence of an infection-associated immune-mediated vasculitis are still speculative. It is currently postulated that bystander activation, microbial persistence, or molecular mimicry [28] and genetic factors [29] concurrently play a crucial role.

## Supplementary Information

Below is the link to the electronic supplementary material.

Supplementary file1 (DOCX 86 KB)
