# Supplementary material for: Infections or Vaccines Associated with Finkelstein-Seidlmayer Vasculitis: Systematic Review
Source: Clin Rev Allergy Immunol. 2022 May 12;63(3):490–8. doi: 10.1007/s12016-022-08940-2 (PMC9096064; doi:10.1007/s12016-022-08940-2)
Supplement: Supplementary file 1 — Supplementary file1 (DOCX 86 KB) [file 12016_2022_8940_MOESM1_ESM.docx]

**Supplementary material**

**Acute hemorrhagic edema of young children – references 1970-2021**

1. Laugier P, Hunziker N, Reiffers J, Rudaz M. L’œdème aigu hémorragique du nourrisson (purpura en cocarde avec oedème). Dermatology 1970;141(2):113–118.
2. Garofalo E. Porpora anafilattoide a coccarda di Seidlmayer. Minerva Pediatr **1972;24(20):817-827.**
3. Verdura G, Lupi G. Porpora vascolare (anafilattoide) acuta in lattante di sei mesi. Confini nosologici della sindrome. Minerva Pediatr 1972;24(13):519-525.
4. Maleville J, Alt J, Grosshans E, Heid EM, Storbel M. Oedème aigu hémorragique de la peau du nourrisson: vascularite allergique dermique. À propos d’un nouveau cas. Bull Soc Fr Dermatol Syphiligr 1973;80:432-435.
5. Snoussi N, Strobel M, Heid F, Grosshans E, Malleville J. Oedème aigu hémorragique du nourrisson: démonstration d'une vascularite allergique dermique. Arch Belg Dermatol 1973;29(3):259-260.
6. Carton FX, Piussan C, Denoeux JP. Oedème aigu hémorragique de la peau du nourrisson. Bull Soc Fr Dermatol Syphiligr 1974;81:233–234.
7. Larrègue M, Lesage B, Rossier A. Edema agudo hemorragico del lactante (EAHL) (purpura en escarapela con edema postinfeccioso de Seidlmayer) y vascularitis alergica. Med Cutan 1974;11(3):165-174.
8. Lesage B, Larrègue M, Bouillet F, Rossier A. Oedème aigu hémorragique du nourrisson (purpura en cocarde avec oedème post-infectieux de Seidlmayer) et vascularité allergique. Ann Pédiatr (Paris) 1975;22(8-9):599-606.
9. Castel Y, Massé R, Le Fur JM, Alix D, Herry B, Olivre MA. L’oedème aigu hémorragique de la peau du nourrisson: étude clinique et nosologique. Ann Pédiatr (Paris) 1976;23(10):653–666.
10. Levi L, Bellini S, Galbiati G, Fontana M. L’edema acuto emorragico della pelle del lattante. Chron Dermatol 1976;7(5):619-621.
11. Despert F, Fauchier C, Laugier J. L’oedeme aigue hemorragique du nourrison : à propos d’une observation. Rev Med Tours 1977;11(5):729-732.
12. Pierini DO, García Diaz R, Pierini AM. Edema agudo hemorrágico del lactante. Púrpura en escarapela. Arch Argent Dermatol 1977;27(1):19-31.
13. Ruhrmann G. Kokardenpurpura und Hämorrhagische Variante des Erythema exsudativum multiforme als Folge einer Mykoplasma-pneumoniae-Infektion? Pädiatr Prax 1977;19:37-40.
14. Lambert D, Laurent R, Bouilly D, Saint-Mont C, Estavoyer JM, Agache P, Chapuis JL. Oedème aigu hémorragique du nourrisson. Données immunologiques et ultrastructurales. Ann Dermatol Venereol 1979;106(12):975-987.
15. Cottoni F, Cambosu GA. Edema emorragico acuto del lattante. Osservazioni su due casi. Ann Ital Dermatol Clin Sper 1980; 34:169-175.
16. Larrègue M, Lorette G, Prigent F, Canuel C. Oedème aigu hémorragique du nourrisson avec complication léthale digestive. Ann Dermatol Venereol 1980;107(10):901-905.
17. Bianchi G, Giannattasio G, Lietti D, Palma D, Peverelli GP, Zanini R. L’edema acuto emorragico del lattante - Descrizione di un caso clinico. Minerva Pediatr 1981;33(2):87-90.
18. Saggese M. L'edema emorragico acuto del lattante. Pediatria (Napoli) 1981;89(4):775-785.
19. Waskerwitz S, Christoffel KK, Hauger S. Hypersensitivity vasculitis presenting as suspected child abuse: Case report and literature review. Pediatrics 1981;67(2):283-284.
20. Conte GF, Colombo P, Ronconi C, Cattaneo AM. Porpora di Seidlmayer: contributo clinico. Clin Pediatr (Bologna) 1982;64:43-48.
21. Sánchez-del-Río J, Marín MC, Martínez DN. Edema agudo hemorragico del lactante. Actas Dermosifiliogr 1982;73(3-4):129-132.
22. Berger C. Oedème aigu hémorragique de la peau du nourrisson: étude gènèrale à propos d’une observation. Lyon Méd 1984;252:35-38.
23. Schiuma AA, Gelmetti C, Cerri D, Gianotti F. Purpura de Finkelstein (edema agudo hemorrágico del lactante). Arch Argent Dermatol 1984;34(5):241-249.
24. Jeannoel P, Fabre M, Payen C, Bost M. Oedème aigu hémorragique du nourrisson: rôle de l’adénovirus? Á propos d’une observation. Pédiatrie 1985;40(7):557-560.
25. Jiménez D, Armijo R, Burkhardt P, Naranjo R. Edema agudo hemorrágico del lactante. Actas Dermosifiliogr 1985;76(11-12):529-531.
26. Morelli P, Della Morte MA, Silva A, Valli F. La porpora ”a coccarda” di Seidlmayer - Presentazione di un caso. Pediatr Med Chir 1985;7(2):325-329.
27. Brown J, Melinkovich P. Schönlein-Henoch purpura misdiagnosed as suspected child abuse. A case report and literature review. JAMA 1986;256(5):617-618.
28. Le Berre A, Plantin P, Metz C, Guillet G. Oedème aigu hémorragique du nourisson: à propos d’un cas - Discussion de l’intérêt du dosage du facteur XIII de la coagulation. Nouv Dermatol 1987;6(3):273-276.
29. Neri I, Patrizi A, Costa AM, Pizzino D, Chiodo D. Quattro casi di edema emorragico acuto del lattante. Pediatr Dermatol News 1987;7:107-110.
30. Stögmann W, Blümel P. Kokardenpurpura: a special type of hypersensitivity vasculitis. Pediatr Dermatol News 1987;6:101–103.
31. Dubin BA, Bronson DM, Eng AM. Acute hemorrhagic edema of childhood: an unusual variant of leukocytoclastic vasculitis. J Am Acad Dermatol 1990;23(2 Pt 2):347-350. doi: 10.1016/0190-9622(90)70219-8.
32. Hirschel-Scholz S, Hunziker N. Acute hemorrhagic edema of the infant (Finkelstein's disease). Pediatr Dermatol 1990;7(4):323. doi: 10.1111/j.1525-1470.1990.tb01038.x.
33. Orozco Covarrubias ML, Laterza Gambuti AM, Tamayo Sánchez L, Ruiz-Maldonado R. Edema agudo hemorrágico del lactante (púrpura en escarapela). Med Cutan Ibero Lat Am 1990;18:392-396.
34. Saraçlar Y, Tinaztepe K, Adalioğlu G, Tuncer A. Acute hemorrhagic edema of infancy (AHEI) - a variant of Henoch-Schönlein purpura or a distinct clinical entity? J Allergy Clin Immunol 1990;86(4 Pt 1):473-483. doi: 10.1016/s0091-6749(05)80202-7.
35. Amoric JC, Stalder JF, Litoux P. Quel est votre diagnostic? Oedème aigu hémorragique du nourrisson. Ann Dermatol Venereol 1991;118(6-7):481-482.
36. Gorgojo Lopéz M, Vélez Garcia-Neto A, López-Barrantes V, González Mediano I, Zambrano Zambrano A. Edema agudo hemorrágico del lactante. Actas Dermosifiliogr 1991;82(10):648-652.
37. Legrain V, Lejean S, Taïeb A, Guillard JM, Battin J, Maleville J. Infantile acute hemorrhagic edema of the skin: study of ten cases. J Am Acad Dermatol 1991;24(1):17-22. doi: 10.1016/0190-9622(91)70002-j.
38. Brunner M, Stieh J. Infantiles akutes hämorrhagisches Ödem. Z Hautkr 1992; 67(5):458-459.
39. Cox NH. Seidlmayer’s syndrome: postinfectious cockade purpura of early childhood. J Am Acad Dermatol 1992;26(2 Pt1):275. doi: 10.1016/s0190-9622(08)80314-3.
40. Sipahi T, Yöney A, Tuna F, Karademir S. Acute hemorrhagic edema of infancy. Anatol J Pediatr 1992;2:74-76.
41. Vandeghinste N, Naeyaert JM, Geerts ML, Kint A. Das akute hämorrhagische Ödem beim Säugling. Hautarzt 1992;43(12):786-788.
42. Amitai Y, Gillis D, Wasserman D, Kochman RH. Henoch-Schönlein purpura in infants. Pediatrics 1993;92(6):865-867.
43. Crespi HG, Ibarra PM, Porta JA. Edema agudo hemorrágico del lactante (Púrpura de Finkelstein). Rev Argent Dermatol 1993;74:54-58.
44. Echeverría Arellano A, Vives Nadal R, Romero Ibarra C, Guarch Troyas R, Durán Urdaniz G. Edema agudo hemorrágico del lactante. An Esp Pediatr 1993;39(5):448-450.
45. Yeste Fernandez D, Gonzalez Castro U, Gonzalez Morla J, de Mir Messa I, Martin Gonzalez M, Castello Girona F. Edema agudo hemorrágico del lactante. An Esp Pediatr 1993;38(1):79-81.
46. Alcalde Alonso M, Juárez Morales E, Daza Torres A, López Muñoz J, Olivar Buera M. Edema agudo hemorrágico del lactante. Actas Dermosifiliogr 1994;85(3):154-156.
47. García Calatayud S, Lozano de la Torre JM, de las Cuevas Terán I, Mirones Martínez Y, Fernández Llaca H. Edema agudo hemorrágico del lactante. Aportación de dos nuevos casos. Bol Pediatr 1994;35:339-343.
48. Garcia-Patos V, Pujol RM, Qureol J, Solis MJ, Oliva E, De Moragas JM. Edema agudo hemorrágico del lactante. Med Cutan Iber Lat Am 1994;22(3):151-154.
49. Kainz JT, Smolle J. Frühinfantile, postinfektiöse Kokarden-Purpura Seidlmayer (Akutes hämorrhagisches Ödem beim Säugling). Z Hautkrankh 1994;69(8):547-548.
50. Al-Sheyyab M, El-Shanti H, Ajlouni S, Sawalha D, Daoud A. The clinical spectrum of Henoch-Schönlein purpura in infants and young children. Eur J Pediatr 1995; 154(12): 969-972. doi:10.1007/BF01958639.
51. Barbaud A, Gillet-Terver MN, Schmutz JL, Weber M. Cas pour diagnostic: oedème aigu hémorragique du nourrisson. Ann Dermatol Venereol 1995;122:717-719.
52. Çaliskan S, Tasdan Y, Kasapçopur Ö, Sever L, Tunnessen WW. Picture of the month: acute hemorrhagic edema of infancy. Arch Pediatr Adolesc Med 1995;149(11):1267-1268. doi: 10.1001/archpedi.1995.02170240085014.
53. Ince E, Mumcu Y, Suskan E, Yalcinkaya F, Tümer N, Cin S. Infantile acute hemorrhagic edema: a variant of leukocytoclastic vasculitis. Pediatr Dermatol 1995;12(3):224-227. doi: 10.1111/j.1525-1470.1995.tb00163.x.
54. Lazarov A, Attias O, Avinoach I, Halevy S. Infantile acute haemorrhagic edema following vaccine application. J Eur Acad Dermatol Venereol 1995;5(2):203-204.
55. Pride HB, Maroon M, Tyler WB. Ecchymoses and edema in a 4-month-old boy. Pediatr Dermatol 1995;12(4):373-375. doi: 10.1111/j.1525-1470.1995.tb00206.x
56. Criado PR, Sakai Valente NY, Jardim Criado RF, Sittart JAS, Sawaya S. Edema agudo hemorrágico do lactente. An Bras Dermatol 1996;71(5):403-406.
57. Cunningham BB, Caro WA, Eramo LR. Neonatal acute hemorrhagic edema of childhood: case report and review of the English-language literature. Pediatr Dermatol 1996;13(1):39-44. doi: 10.1111/j.1525-1470.1996.tb01186.x.
58. Krause I, Lazarov A, Rachmel A, Grunwald MM, Metzker A, Garty BZ, Halevy S, Nitzan M. Acute haemorrhagic oedema of infancy, a benign variant of leucocytoclastic vasculitis. Acta Paediatr 1996;85(1):114-117. doi: 10.1111/j.1651-2227.1996.tb13904.x.
59. Lantner RR, Simon PR. Acute hemorrhagic edema of infancy. Pediatr Emerg Care 1996;12(2):111-112. doi: 10.1097/00006565-199604000-00012.
60. Mássimo JA, Giglio N, Cotton A, Goldfarb G. Cual es su diagnostico? Edema agudo hemorragico del lactante. Rev Hosp Ninos 1996;38(4):287-288.
61. Tomaç N, Saraçlar Y, Türktas I, Kalayci Ö. Acute haemorrhagic oedema of infancy: a case report. Clin Exp Dermatol 1996;21(3):217-219. doi:10.1111/j.1365-2230.1996.tb00067.x.
62. Colantonio G, Kinzlansky V, Kahn A, Damilano G. Pathological case of the month: infantile acute hemorrhagic edema. Arch Pediatr Adolesc Med 1997;151(5):523-524. doi: 10.1001/archpedi.1997.02170420093018
63. Gattorno M, Picco P, Gambini C, Buoncompagni A, Castagnola E, Pistoia V, Borrone C. Erythema multiforme-like manifestations and arthritis in a 3-year-old child with leukocytoclastic vasculitis. Clin Exp Rheumatol 1997;15(3):329-332.
64. Scaramuzza A, Pezzarossa E, Zambelloni C, Lupi A, Lazzari GB, Rossoni R. Case of the month: a girl with oedema and purpuric eruption. Diagnosis: acute haemorrhagic oedema of infancy. Eur J Pediatr 1997;156(10):813-815. doi: 10.1007/s004310050720.
65. Aydın K, Kendirci M, Utaş S, Üstünbaş HB, Kısaarslan AF. Acute hemorrhagic edema of infancy: A case report with mucosal and truncal involvement. Turkiye Klinikleri J Pediatr 1998;7:93-95.
66. Cabrera Roca G, Domínguez Ortega F, Perdomo Mesa E, Medina Calvo F, Báez Marrero O. Edema agudo hemorrágico del lactante: presentación de 5 casos. An Esp Pediatr 1998;48(1):73-75.
67. Castro Piñeiro I, Hernández Santana J, Soler Cruz E, Rodríguez López J, Hernández Hernández B. Edema agudo hemorrágico del lactante. Actas Dermosifiliogr 1998;89:189-93.
68. Cocchi P, Battini ML, Di Maria M, Calabri GB, Masoni F, Piccinini P, Salvatore A, Franchini F. Edema emorragico infantile acuto. Descrizione di un nuovo caso clinico. Pediatr Med Chir 1998;20(3):227-229.
69. Crowe MA, Jonas PP. Acute hemorrhagic edema of infancy. Cutis 1998;62(2):65-66.
70. Gonggryp LA, Todd G. Acute hemorrhagic edema of childhood (AHE). Pediatr Dermatol 1998;15(2):91-96. doi: 10.1046/j.1525-1470.1998.1998015091.x.
71. İnalhan M, Sümer B, Sarper N, Şener B, Yılmaz S, İnan S. A case infantile acute hemorrhagic edema. Zeynep Kamil Tıp Bülteni 1998;30(3-4):179-183.
72. Long D, Helm KF. Acute hemorrhagic edema of infancy: Finkelstein’s disease. Cutis 1998;61:283-284.
73. Ricci G, Patrizi A, Specchia F, Neri I, Masi M. Infantile acute haemorrhagic oedema in a child with von Willebrand’s disease. Acta Derm Venereol 1998;78(1):78-79. doi: 10.1080/00015559850135986.
74. Stringa S, Perissé B, Stringa O, Civitillo C. Edema agudo hemorragico del lactante: estudio de dos casos. Dermatol Argent 1998;4(3):273-277.
75. Yang CH, Shih IH, Yang LJ, Chan HL, Kuo TT. Acute hemorrhagic edema of infancy. Dermatol Sinica 1998;16:236-241.
76. Gattorno M, Picco P, Vignola S, Di Rocco M, Buoncompagni A. Brother and sister with different vasculitides. Lancet 1999;353(9154):728. doi: 10.1016/s0140-6736(98)05109-5.
77. Millard T, Harris A, MacDonald D. Acute infantile hemorrhagic oedema. J Am Acad Dermatol 1999;41(5 Pt 2):837-839. doi: 10.1016/s0190-9622(99)70338-5.
78. Morrison RR, Saulsbury FT. Acute hemorrhagic edema of infancy associated with pneumococcal bacteremia. Pediatr Infect Dis J 1999;18:832-833.
79. Uribe-Jiménez E, Guevara-Gutiérrez E, González-Esqueda P, Loza-Vega M, Tlacuilo-Parra A, Gómez-Guerrero R. Edema hemorrágico agudo de la infancia: informe de un caso y revisión de la literatura. Actas Dermosifiliogr 1999;90:575-578.
80. Alonso Falcón F, García Consuegra J. Gastroenteritis por Campylobacter jejuni y edema agudo hemorrágico del lactante. Pediátrika 2000;20(7):254-256.
81. Carbajal RL, Zarco RJ, Rodríguez HR, Reynes MJN, Barrios Fuentes R, Luna FM, Villegas VE. Edema hemorrágico agudo y púrpura de Henoch-Schönlein ¿Son una misma enfermedad en los lactantes? Rev Mex Pediatr 2000;67(6):266-269.
82. Pezzarossa E, Lazzari GB. Edema emorragico acuto infantile (malattia di Finkelstein) e presentazione di due casi clinici. G Ital Dermatol Venereol 2000;135(5):593-596.
83. Shetty AK, Desselle BC, Ey JL, Correa H, Galen WK, Gedalia A. Infantile Henoch-Schönlein purpura. Arch Fam Med 2000;9:553-556.
84. Dönmez O, Memesa A. Akut ýnfantýl hemorajýk ödem: üç olgunun takdýmý. ADÜ Týp Fakültesi Dergisi 2001;2(3):31-33.
85. Fujimura T, Funayama M, Tagami H. Acute hemorrhagic edema in a four-year-old Japanese boy. J Dermatol 2001;28:279-281.
86. Govoni MR, Strumia R, De Rosa E, Malagutti L, De Sanctis V. Edema emorragico acuto dell’infanzia. Presentazione di un caso clinico. Ital J Pediatr 2001;27:811-813.
87. Guerrero Fernández J, Guiote Domínguez MV, Guerrero Vázquez J, García Sicilia J, Rodríguez Delgado J. Edema hemorrágico agudo del lactante. A propósito de tres casos. Ciencia Pediátrika 2001;21(1):26-30.
88. Jacobzone C, Plantin-Eon I, Vic P, Broussine L, Plantin P. Plaidoyer pour renommer l'oedème aigu hémorragique du nourrisson. Arch Pédiatr 2001;8(7):770-771. doi: 10.1016/s0929-693x(00)90314-2.
89. Jayanti S, Srinivas M, Marwaha RK, Trehan A. Leucocytoclastic vasculitis in infant. Indian J Pediatr 2001;68(5):457-458. doi: 10.1007/BF02723028.
90. Offidani A, Cellini A, Bossi G. Guess what! Acute haemorrhagic oedema of the skin in infancy. Eur J Dermatol 2001;11:63-64.
91. Paradisi M, Annessi G, Corrado A. Infantile acute hemorrhagic edema of the skin. Cutis 2001;68:127-129.
92. Slee DSJ, Lagro SWY, Frenkel J. Acuut hemorragisch oedeem bij kinderen: uitstekende prognose. Ned Tijdschr Geneeskd 2001;145(17):830-834.
93. Vermeer MH, Stoof TJ, Kozel MMA, Blom DJM, Nieboer† C, Sillevis Smitt JH. Acuut hemorragisch oedeem van de kinderleeftijd en het onderscheid met Henoch-Schönlein-purpura. Ned Tijdschr Geneeskd 2001;145(17):834-839.
94. Ballona R. Pápulas purpúricas en récien nacido. Dermatol Peru 2002;12:231-233.
95. Bozaykut A, Atay E, Atay Z, Ipek Ö, Akin M, Dursun E. Acute infantile haemorrhagic oedema associated with hepatitis A. Ann Trop Paediatr 2002;22(1):59-61. doi: 10.1179/027249302125000175.
96. Braun-Falco M, Abeck D. Acute infantile haemorrhagic oedema. Lancet 2002;360:210. doi: 10.1016/S0140-6736(02)09457-6.
97. Çaksen H, Odabas D, Kösem M, Arslan S, Öner AF, Atas B, Akçay G, Ceylan N. Report of eight infants with acute infantile hemorrhagic edema and review of the literature. J Dermatol 2002;29(5):290-295.
98. Escobosa Sánchez OM, Chica Fuentes YM, Ranchal Pérez P, Jiménez Hinojosa JM, Blasco Alonso J, Madrid Madrid A, Durán Hidalgo I, Jurado Ortiz A. Edema agudo hemorrágico de la infancia (enfermedad de Finkelstein). An Esp Pediatr 2002;56(Suppl 5):110.
99. Garty BZ, Ofer I, Finkelstein Y. Acute hemorrhagic edema of infancy. Isr Med Assoc J 2002;4:228-229.
100. Gauger A, Braun-Falco M, Stachowitz S, Ring J, Abeck D. Akut aufgetretene ödematöse Ekchymosen beim Kleinkind. Hautarzt 2002;53:559-560. doi: 10.1007/s00105-002-0399-8.
101. Kuroda K, Yabunami H, Hisanaga Y. Acute haemorrhagic oedema of infancy associated with cytomegalovirus infection. Br J Dermatol 2002;147(6):1254-1257. doi: 10.1046/j.1365-2133.2002.05038.x. PMID: 12452880.
102. Miorin E, Meneghini A, Don B, Romanello C, Tenore A. Edema emorragico acuto del lattante, descrizione di un caso clinico e revisione della letteratura. Medico e Bambino pagine elettroniche 2002;5(3) https://www.medicoebambino.com/?id=CL0203_10.html Last access January 2020.
103. Montesinos Sanchís E, Rodríguez Varela A, Pons Fernández N, Pérez Monjardín E, Rodríguez B, Mercader P, Ardit J, Álvarez V. Edema agudo hemorrágico del lactante. An Esp Pediatr 2002;56(Suppl 5):158.
104. Saray Y, Seçkin D, Sarifakioglu E, Güleç AT, Demirhan B. Acute infantile haemorrhagic oedema: measles vaccination as possible triggering factor. Acta Derm Venereol 2002;82(6-7):471-472.
105. Shah D, Goraya JS, Poddar B, Parmar VR. Acute infantile hemorrhagic edema and Henoch-Schönlein purpura overlap in a child. Pediatr Dermatol 2002;19(1):92-93. doi: 10.1046/j.1525-1470.2002.0024d.x.
106. Barkai G, Sinai L. Index of suspicion. Pediatr Rev 2003;24(10):349-354.
107. Guerra Tapia A, González E, Rodríguez Peralto JL. Diagnósticos clínicos. Púrpura en escarapela en un lactante. Actualidad dermatológica: revista científica de dermatología médico-quirúrgica, 2003;42(7):617-619.
108. Kaur S, Thami GP. Urticarial vasculitis in infancy. Indian J Dermatol Venereol Leprol 2003;69:223-224.
109. La Placa G, Pradella C, Andreotti M, Befana R. Edema emorragico acuto dell’infanzia. Descrizione di un caso clinico. Pediatr Med Chir 2003;25(1):60-62.
110. Lakshmi C, Srinivas CR. Urticarial vasculitis of infancy (acute hemorrhagic edema). Indian J Dermatol Venereol Leprol 2003;69(6):427.
111. Macèa JM, Santi CG, Sotto MN, Caputo R. Multiple erythematous plaques on a child. Acute hemorrhagic edema of infancy. Arch Dermatol 2003;139:531-536.
112. Poyrazoglu HM, Per H, Guündüz Z, Düsünsel R, Arslan D, Narin N, Gümüs H. Acute hemorrhagic edema of infancy. Pediatr Int 2003;45:697-700.
113. Smoje G, Cuevas M, Núñez L, Bolte C, Martínez W, Henríquez A. Edema hemorrágico agudo de la infanzia: presentación de un caso clínico. Rev Chil Pediatr 2003;74:625-26.
114. Britto Goulart F, de Sá Torres Lage K, Quinterno MV, Madureira de Pádua P. Edema agudo hemorrágico da infância. Rev Bras Reumatol 2004;44(3):251-254.
115. da Silva Manzoni APD, Viecili JB, de Andrade CB, Kruse RL, Bakos L, Cestari TF. Acute hemorrhagic edema of infancy: a case report. Int J Dermatol 2004;43:48-51.
116. Di Lernia V, Lombardi M, Lo Scocco G. Infantile acute hemorrhagic edema and rotavirus infection. Pediatr Dermatol 2004;21(5):548-550.
117. Goldaracena P, Peréz F. Edema agudo hemorrágico del lactante. Arch Argent Pediatr 2004;102(1):72-73.
118. Miner I, Muñoz JA, Landa J, Albisu Y. Edema agudo hemorrágico del lactante. An Pediatr (Barc) 2004;61(1):79-81.
119. Rodrigues F, Coelho S. Edema agudo hemorrágico do lactente. Acta Pediátr Port. 2004;35(2):149-151.
120. Roh MR, Chung HJ, Lee JH. A case of acute hemorrhagic edema of infancy. Yonsei Med J 2004;45(3):523-526.
121. Wong CT, Harrington JW. Infantile Henoch-Schönlein purpura. Emerg Med Australas 2004;16:225-228. doi: 10.1111/j.1742-6723.2004.00591.x.
122. Acar B, Özcakar ZB, Yüksel S, Pekacar T, Ekim M, Yalcinkaya F. A 12-month-old boy with high fever, erythematous lesions and haemorrhagic oedema. Eur J Pediatr 2005;164:453-454. doi: 10.1007/s00431-005-1641-z.
123. Bozaykut A, Pulat-Seren L, Özahi-İpek İ, Vardar-Tuncel G. Acute infantile hemorrhagic edema: report of three cases. Çocuk Sağlığı ve Hastalıkları Dergisi. 2005; 48(2):147-150.
124. Emerich P, Neves A, Machado I, Fagundes S, Almeida P. Edema agudo hemorrágico de la infanzia. Dermatol Pediatr Lat 2005;3(3):234-238.
125. García Lorenzo R, Alca´zar Lozano C, Fernández Alonso JE, Urueña Leal C, Cancho Candela R, Miranda Fontes M. Edema agudo hemorrágico del lactante. Bol Pediatr 2005;45(192):136-137.
126. Martín Hernández JM, Jordá Cuevas E, Monteagudo de Castro C, Alonso Usero V, Pereda Carrasco C, Ramón Quilez D. Edema agudo hemorrágico del lactante. Piel 2005;20(4):167-171.
127. McDougall CM, Ismail SK, Ormerod A. Acute haemorrhagic oedema of infancy. Arch Dis Child 2005;90:316. doi: 10.1136/adc.2004.060632.
128. Acun C, Ustundag G, Sogut A, Koca R, Numanoglu G. Visual diagnosis: a child who has acute onset of unusual skin lesions and edema. Pediatr Rev 2006;27(11):e71-e74.
129. Araníbar D, Giacaman S, Villagrán V, Saavedra U. Edema hemorrágico agudo del lactante: reporte de dos casos. Rev Chil Dermatol 2006;22(4):262-266.
130. Can B, Kavala M, Türkoglu Z, Zemheri E. Acute hemorrhagic edema of infancy: a case report. Turk J Pediatr 2006;48:266-268.
131. Garty BZ, Pollak U, Scheuerman O, Marcus N, Hoffer V. Acute hemorrhagic edema of infancy associated with herpes simplex type 1 stomatitis. Pediatr Dermatol 2006;23(4):361-364.
132. Gaslini G. Un lattante con edema al volto e una strana porpora. Riviste Digitali: Il Pediatra 2006;5:36-37.
133. Gomes Silveira JC, Lobato Quattrino A, Bragança R, Rochael MC. Edema hemorrágico agudo de infância. An Bras Dermatol 2006;81(Suppl 3):S285-S287.
134. Lee HM, Kang EY, Kim HU, Hwang PH. Acute hemorrhagic edema in an infant mimicking Henoch-Schönlein purpura: a case study. Korean J Pediatr 2006;49(12):1354-1357.
135. Lilian Pérez C, Alicia Benavides M, Bárbara Barrientos F, Cristian Deza E, Cristó bal Guixe A, Gonzalo Mendoza L. Edema hemorrágico agudo del lactante. Rev Chil Pediatr 2006;77(6):599-603.
136. Liu AJW, Hogan P, Nanan R. Acute haemorrhagic oedema of infancy. Arch Dis Child 2006;91:382. doi: 10.1136/adc.2005.093286.
137. Mata Fernández C. Edema hemorrágico agudo del lactante: una entidad poco conocida. Rev Pediatr Aten Primaria 2006; 8(31):435-438.
138. Michael DJ. Acute hemorrhagic edema of infancy. Dermatol Online J 2006;12(5):10.
139. Pereira T, Nunes S, Vieira AP, Sá A, Sousa Basto A. Edema hemorrágico agudo infantil. Trab Soc Port Dermatol Venereol 2006;64(2):243-250.
140. Pérez Fernández F, García Aldána D, Torres Borrego J, Luque Moreno M. Edema agudo hemorrágico infantil. Experiencia con cinco casos. Acta Pediátr Esp 2006;64(8):399-401.
141. Zamberk Majlis P, Velázquez Tarjuelo D, Campos Domínguez M, Leis Dosi VM, Hernanz Hermosa JM. Edema hemorrágico del lactante. Acta Pediatr Esp 2006;64(11):551-553.
142. Blasini W, Saini R, Vincek V. Acute hemorrhagic edema of infancy: a case report. Dermatol Online J 2007;13(3):37.
143. Chatproedprai S, Wananukul S. Acute hemorrhagic edema of infancy. J Med Assoc Thai 2007;90(6):1205-1207.
144. Domínguez LM, Aldama O, Rivelli V, Gorostiaga G, Mendoza G, Aldama A. Edema agudo hemorrágico del lactante. Reporte de un caso. Dermatol Pediatr Lat 2007;5(2):121-124.
145. Ensslen M, Messer G, Liese J. Purpura und bilaterale Ohrschwellung. Monatsschr Kinderheilkd 2007;155:686. doi: 10.1007/s00112-007-1542-5.
146. Epçaçan SOM, Tuncer O, Doğan M, Çaksen H. Acute infantile hemorrhagic edema in differential diagnosis of purpura. Van Med J 2007;14(1):31-34.
147. Kulcu NU, Degirmenci S, Arman D, Guven F, & Say A. Case Report: Acute Hemorrhagic Edema of Infancy. Journal of pediatric infection 2007;1(1):33-35.
148. Kurugol Z, Ozdemir R, Turkoglu E. Infantile Henoch-Schönlein purpura. Pediatr Int 2007;49:680-682. doi: 10.1111/j.1442-200X.2007.02440.x.
149. Lai-Cheong JE, Banerjee P, Hill V, Kenny P, Ross J. Bullous acute haemorrhagic oedema of skin in infancy. Clin Exp Dermatol 2007;32:467-468. doi: 10.1111/j.1365-2230.2007.02355.x.
150. Paredes N, Rubio R, Silva S. Edema hemorrágico agudo del lactante: a propósito de un caso. Dermatol Pediatr Lat 2007; 5(3):182-185.
151. Pelajo C, de Oliveira S. Edema hemorrágico agudo da infância e uma variante da púrpura de Henoch-Schönlein? Rev Bras Reumatol 2007;47(1):69-71.
152. Suehiro RM, Soares BS, Eisencraft AP, Campos LM, Silva CA. Acute hemorrhagic edema of childhood. Turk J Pediatr 2007; 49:189-192.
153. Watanabe T, Sato Y. Renal involvement and hypocomplementemia in a patient with acute hemorrhagic edema of infancy. Pediatr Nephrol 2007;22(11):1979-1981. doi: 10.1007/s00467-007-0599-7.
154. Yilmazo Ş, Dagdemir A, Gökçe IK, Süllü Y, Kiliniç AA. Aktif infantil Hemorajik Ödem: Olgu Sunumu. M.Ü. Tip Dergisi 2007;24(4):137-140.
155. Yu JE, Mancini AJ, Miller ML. Intussusception in an infant with acute hemorrhagic edema of infancy. Pediatr Dermatol 2007;24(1):61-64. doi: 10.1111/j.1525-1470.2007.00336.x.
156. Babić S, Murat-Sušić S, Husar K, Skerlev M, Radoš J. Acute hemorrhagic edema of infancy: case report. Acta Dermatovenerol Croat 2008;16(2):87-90.
157. Çakçak DS, Çakçak B, Akman A, Güney SV, Başsorgun Cİ, Çiftçioğlu MA. Purpuranın ayırıcı tanısında yer alan akut infantil hemorajik ödem olgusu. Türk Dermatoloji Dergisi 2008;2:84-86.
158. Cicero M. Rash decisions: acute hemorrhagic edema of infancy in a 7-month-old boy. Pediatr Emerg Care 2008;24(7):501-502.
159. Fiore E, Rizzi M, Ragazzi M, Vanoni F, Bernasconi M, Bianchetti MG, Simonetti GD. Acute hemorrhagic edema of young children (cockade purpura and edema): a case series and systematic review. J Am Acad Dermatol 2008;59(4):684-695. doi: 10.1016/j.jaad.2008.06.005.
160. Jain G, Patel A. Acute hemorrhagic edema of infancy. Indian Pediatr 2008;45(10):866-867.
161. Javidi Z, Maleki M, Mashayekhi V, Tayebi-Maybodi N, Nahidi Y. Acute hemorrhagic edema of infancy. Arch Iran Med 2008;11(1):103-106.
162. [Kumar R](https://www.ncbi.nlm.nih.gov/pubmed/?term=Kumar%20R%5BAuthor%5D&cauthor=true&cauthor_uid=19129571), [Mittal K](https://www.ncbi.nlm.nih.gov/pubmed/?term=Mittal%20K%5BAuthor%5D&cauthor=true&cauthor_uid=19129571), [Rawal M](https://www.ncbi.nlm.nih.gov/pubmed/?term=Rawal%20M%5BAuthor%5D&cauthor=true&cauthor_uid=19129571), [Kumar S](https://www.ncbi.nlm.nih.gov/pubmed/?term=Kumar%20S%5BAuthor%5D&cauthor=true&cauthor_uid=19129571). Acute hemorrhagic edema of infancy. Indian Pediatr 2008;45(12):1002-1003.
163. Obeid M, Haley J, Crews J, Parhizgar R, Johnson L, Camp T. Acute hemorrhagic edema of infancy with abdominal pain and elevated transaminases. Pediatr Dermatol 2008;25(6):640-641. doi: 10.1111/j.1525-1470.2008.00791.x.
164. Sites LY, Woodmansee CS, Wilkin NK, Hanson JW, Skinner RB Jr, Shimek CM. Acute hemorrhagic edema of infancy: case reports and a review of the literature. Cutis 2008;82(5):320-324.
165. Alp H, Artaç H, Alp E, Reisli I. Acute infantile hemorrhagic edema: a clinical perspective (report of seven cases). Marmara Med J 2009;22:155–161.
166. AlSufyani MA. Acute hemorrhagic edema of infancy: unusual scarring and review of the English language literature. Int J Dermatol 2009;48(6):617-622. doi: 10.1111/j.1365-4632.2009.03917.x.
167. Chaudhry SH, Both H, Versteeg J, van Praag M. Infant with tender oedema and purpuric lesions (Case presentation). Acta Paediatr 2009;98(9):1390-1391, 1537-1538. doi: 10.1111/j.1651-2227.2009.01380.x.
168. El Hafidi N, [Allouch B](javascript:void(0);), Benbrahim F, Mahraoui C. L'oedème aigu hémorragique du nourrisson: une vascularite bénigne et récidivante. J Pediatr Pueric 2009;22(4-5):202-204. doi:10.1016/j.jpp.2009.03.004.
169. Ergüven M, Karaca Atakan S. Infantile hemorrhagic edema due to parvovirus B19 infection. J Child 2009;9(1):43-45.
170. Floristán Muruzabal U, Moreno Alonso de Celada R, Feltes Ochoa RA, Álvarez Gil N, De Lucas Laguna R. Edema agudo hemorrágico de la infancia: un trastorno de curso benigno. Rev Esp Pediatr 2009;65(2):126-128.
171. Karremann M, Jordan AJ, Bell N, Witsch M, Dürken M. Acute hemorrhagic edema of infancy: report of 4 cases and review of the current literature. Clin Pediatr (Phila). 2009;48(3):323-326. doi: 10.1177/0009922808323113.
172. Menteş SE, Taşkesen M, Katar S, Günel ME, Akdeniz S. Acute hemorrhagic edema of infancy. Dicle Univ Tip Fakul Derg 2009;36(1):56-58.
173. Niccoli AA, Castellani MS, Gerardini E, Fioretti P, Castellucci G. Edema emorragico acuto del lattante: descrizione di un caso clinico. Riv Ital Pediatr Osped 2009;2(3):23-25.
174. Tagliabue A, Bettinelli A, Cogliati F. Edema acuto emorragico della prima infanzia (Porpora di Seidlmayer). Medico e Bambino pagine elettroniche 2009;12(6) https://www.medicoebambino.com/?id=CL0906_40.html.
175. Valencia-Herrera AM, Morales-Fuentes RA, Mejía-Rodríguez SA, Álvarez-Reyes A, Moguel-Parra G, Escobar-Sánchez A, Mena-Cedillos CA. Infantile acute hemorrhagic edema in a female child: a different entity from Henoch-Shönlein purpura. Bol Med Hosp Infant Mex 2009;66(5):440-445.
176. [Abbas O](https://www.ncbi.nlm.nih.gov/pubmed/?term=Abbas%20O%5BAuthor%5D&cauthor=true&cauthor_uid=20631278), [Ghosn S](https://www.ncbi.nlm.nih.gov/pubmed/?term=Ghosn%20S%5BAuthor%5D&cauthor=true&cauthor_uid=20631278). Question: Can you identify this condition? Acute hemorrhagic edema of infancy. [Can Fam Physician](https://www.ncbi.nlm.nih.gov/pubmed/20631278) 2010;56(7):666.
177. Halicioglu O, Akman SA, Sen S, Sutcuoglu S, Bayol U, Karci H. Acute hemorrhagic edema of infancy: a case report. Pediatr Dermatol 2010;27(2):214-215. doi: 10.1111/j.1525-1470.2009.00943.x.
178. Kafaie P, Akaberi AA, Hajihossieni H, Taghi Noorbala M. Acute hemorrhagic edema of infancy: A case report. J Pak Assoc Dermatol 2010;20:172-175.
179. Küçüktaşçi K, Semiz S, Çolpan A. Acute hemorrhagic edema of infancy. Turkish J Pediatr Dis 2010;4(2):109-113.
180. Kumar P, Mondal A, Ghosh K, Gharami RC. Acute hemorrhagic edema of infancy with extensive necrosis - a rare presentation. J Turk Acad Dermatol 2010;4(2):04201c.
181. Monteiro C, Lira S, Zilhão C. Edema hemorrágico agudo da infância - Dois casos clínicos. Birth Growth Med J 2010;19(1):14-16.
182. Roldán Ros AM, Rueda Muñoz A, López Lorite AM, Pina Sánchez-Arjona B. Edema hemorrágico agudo en un lactante. Acta Pediatr Esp 2010;68(7):366-368.
183. Slutsky JB, Lee K, Adams CW, Jones EC, Hayman RT. Acral petechiae and purpuric plaques in a 3-year-old girl-quiz case. Acute hemorrhagic edema of infancy (AHEI), or Finkelstein disease. Arch Dermatol 2010;146(9):1037-1042. doi: 10.1001/archdermatol.2010.217-a.
184. Stewart LC, Leech SN, Ulmann D, Sloan P, Abinun M. Acute haemorrhagic oedema of infancy - a case of benign cutaneous leucocytoclastic vasculitis. Rheumatology (Oxford) 2010;49(8):1604-1606. doi: 10.1093/rheumatology/keq104.
185. Bansal S, Ghate S, Jerajani HR. Sudden onset purpura in a healthy infant: acute hemorrhagic edema of infancy. Indian J Dermatol 2011;56(3):349–351.
186. Cacharrón Caramés T, Díaz Soto R, Suárez García F, Rodríguez Valcárcel G. Edema hemorrágico agudo del lactante. An Pediatr 2011;74(4):272-273. doi: 10.1016/j.anpedi.2010.10.024.
187. [Chandrakasan S](https://www.ncbi.nlm.nih.gov/pubmed/?term=Chandrakasan%20S%5BAuthor%5D&cauthor=true&cauthor_uid=21488159), [Singh S](https://www.ncbi.nlm.nih.gov/pubmed/?term=Singh%20S%5BAuthor%5D&cauthor=true&cauthor_uid=21488159), [Dogra S](https://www.ncbi.nlm.nih.gov/pubmed/?term=Dogra%20S%5BAuthor%5D&cauthor=true&cauthor_uid=21488159), [Delaunay J](https://www.ncbi.nlm.nih.gov/pubmed/?term=Delaunay%20J%5BAuthor%5D&cauthor=true&cauthor_uid=21488159), Proust A, [Minz RW](https://www.ncbi.nlm.nih.gov/pubmed/?term=Minz%20RW%5BAuthor%5D&cauthor=true&cauthor_uid=21488159). Wiskott-Aldrich syndrome presenting with early onset recurrent acute hemorrhagic edema and hyperostosis. Pediatr Blood Cancer 2011;56(7):1130-1132. doi: 10.1002/pbc.22888.
188. [Emerich PS](https://www.ncbi.nlm.nih.gov/pubmed/?term=Emerich%20PS%5BAuthor%5D&cauthor=true&cauthor_uid=22281909), [Prebianchi PA](https://www.ncbi.nlm.nih.gov/pubmed/?term=Prebianchi%20PA%5BAuthor%5D&cauthor=true&cauthor_uid=22281909), [Motta LL](https://www.ncbi.nlm.nih.gov/pubmed/?term=Motta%20LL%5BAuthor%5D&cauthor=true&cauthor_uid=22281909), [Lucas EA](https://www.ncbi.nlm.nih.gov/pubmed/?term=Lucas%20EA%5BAuthor%5D&cauthor=true&cauthor_uid=22281909), [Ferreira LM](https://www.ncbi.nlm.nih.gov/pubmed/?term=Ferreira%20LM%5BAuthor%5D&cauthor=true&cauthor_uid=22281909). Acute hemorrhagic edema of infancy: report of three cases. An Bras Dermatol 2011;86(6):1181-1184. doi: 10.1590/s0365-05962011000600019.
189. Ferreira O, Antunes I, Cruz MJ, Mota A, Bettencourt H, Canelhas Á, Azevedo F. Acute hemorrhagic edema of childhood after H1N1 immunization. Cutan Ocul Toxicol 2011;30(2):167-169. doi: 10.3109/15569527.2010.536797.
190. Ilknur T, Fetil E, Lebe B, Güneş AT. Leukocytoclastic vasculitis presenting as acute hemorrhagic edema in a 21-year-old patient. Int J Dermatol 2011;50(7):860-862. doi: 10.1111/j.1365-4632.2010.04489.x.
191. Moradinejad MH, Entezari P, Mahjoub F, Ziaee V. Acute hemorrhagic edema of infancy; a report of five Iranian infants and review of the literature. Iran J Pediatr 2011;21(1):107-112.
192. Turan H, Turan A. Acute hemorrhagic edema of infancy: a case report. Güncel Pediatri 2011;9(2):50-52.
193. Behmanesh F, Heydarian F, Toosi MB. Acute hemorrhagic edema of infancy: report of two cases report. Iran J Blood Cancer 2012;4(2):93-96.
194. Boos MD, McMahon P, Castelo-Soccio L. Acute onset of a hemorrhagic rash in an otherwise well-appearing infant. J Pediatr 2012;161(6):1175. doi: 10.1016/j.jpeds.2012.06.011.
195. Brett A, Rodrigues F, Salgado M. Lesões purpúricas – que diagnóstico? Acta Pediatr Port 2012;43(4):179-180.

1. [Cabanillas-Becerra](https://www.semanticscholar.org/author/Jacqueline-J-Cabanillas-Becerra/1431177583) J, [Pérez-del Arca](https://www.semanticscholar.org/author/C%C3%A9sar-P%C3%A9rez-del-Arca/108776426) C, [Vera](https://www.semanticscholar.org/author/Cecilia-Vera/29419932) C, Barquinero-Fernández A. Edema agudo hemorrágico del lactante. Dermatol Peru 2012;22(4):182-186.
2. Dongre A, Adhe V, Kothari D, Kardekar S, Khopkar U. Acute hemorrhagic edema of infancy: a report of two cases. Indian J Dermatol Venereol Leprol 2012;78(1):121.
3. Fotis L, Nikorelou S, Lariou MS, Delis D, Stamoyannou L. Acute hemorrhagic edema of infancy: a frightening but benign disease. Clin Pediatr (Phila) 2012;51(4):391-393. doi: 10.1177/0009922810396549.
4. Martínez García MS, Casado Sánchez ML. ¿Púrpura de Schönlein-Henoch o edema agudo hemorrágico del lactante? Rev Pediatr Aten Primaria 2012;14(55):231-233.
5. Paula Yarmuch G, Ximena Chaparro R, Cecilia Fischer S, Christian Haussmann G, Samuel Benveniste D. Edema hemorrágico agudo del lactante: reporte de un caso de presentación atípica. Rev Chil Pediatr 2012;83(5):462-467.
6. Alaygut D, Kılıç SÇ, Gülsever O, Gavas HT, Bostancı PK. Bir olgu nedeni ile akut infantil hemorajik ödem. Cumhuriyet Tıp Derg 2013;35:593-596. doi: 10.7197/1305-0028.1886.
7. Alhammadi AH, Adel A, Hendaus MA. Acute hemorrhagic edema of infancy: a worrisome presentation, but benign course. Clin Cosmet Investig Dermatol 2013;6:197–199. doi: 10.2147/CCID.S51525.
8. Carvalho C, Januário G, Maia P Acute haemorrhagic oedema of infancy. BMJ Case Rep 2013;2013:bcr2012008145. doi: 10.1136/bcr-2012-008145.
9. Franco Soto JV, Delgado A, Deivis J, Lidiz MM, Peñuela O. Enfermedad de Finkelstein. Reporte de un caso. Arch Venez Pueric Pediatr 2013;76(1):24-26.
10. Freitas P, Bygum A. Visual impairment caused by periorbital edema in an infant with acute hemorrhagic edema of infancy. Pediatr Dermatol 2013;30(6):e132-e135. doi: 10.1111/j.1525-1470.2012.01871.x.
11. Garcia C, Sokolova A, de Lurdes Torre M, Amaro C. Edema agudo hemorrágico da infância: relato de caso e comparação com meningococcemia. Rev Port Imunoalergol 2013; 21(3):213-214.
12. Jindal SR, Kura MM. Acute hemorrhagic edema of infancy-a rare entity. Indian Dermatol Online J 2013;4(2):106-108. doi: 10.4103/2229-5178.110630.
13. Rodríguez Estoup MV, Hernández M, Soliani A, Abeldaño A. Edema y lesiones purpúricas en miembros inferiores de un lactante. Dermatol Pediatr Lat 2013;11(2):79-82.
14. Savino F, Lupica MM, Tarasco V, Locatelli E, Viola S, di Montezemolo LC, Coppo P. Acute hemorrhagic edema of infancy: a troubling cutaneous presentation with a self-limiting course. Pediatr Dermatol 2013;30(6):e149-e152. doi: 10.1111/pde.12004.
15. Scheer HS, Weibel L. Sudden purpuriform rash in an infant. JAMA 2013;309(20):2159-2160.
16. Sert A, Yazar A, Odabaş D, Çelik AY. Akut infantil hemorajik ödem: İki olgu sunumu. Van Med J 2013;20(3):162-165.
17. Bachmann Holzinger II, Neuhaus TJ, Portmannb S. Kokardenpurpura, rote Ohren und schmerzende Füsse bei kleinen Kindern. Schweiz Med Forum 2014;14(29–30):545–546.
18. Chowdhoury SR, Ganguly, S, Mukherjee S, Samanta M, Datta K. Acute hemorrhagic edema of infancy. Indian J Pediatr 2014;81(8):811-813. doi: 10.1007/s12098-013-1293-3.
19. Di Lernia V. Mycoplasma pneumoniae: an aetiological agent of acute haemorrhagic oedema of infancy. Australas J Dermatol. 2014;55(4):e69-e70. doi: 10.1111/ajd.12047.
20. Dotan M, Nahum E, Weigl D, Bilavsky E. Compartment syndrome because of acute hemorrhagic edema of infancy: a case report and literature review. J Pediatr Orthop B 2014;23(5):419-421. doi: 10.1097/BPB.0000000000000060.
21. [Dutta A](https://europepmc.org/search?query=AUTH:%20Abhijit%20Dutta), [Ghosh SK](https://europepmc.org/search?query=AUTH:%20Sudip%20Kumar%20Ghosh). Acute hemorrhagic edema of infancy. Indian Pediatr 2014;51(8):677-678.
22. Glamann JA, Morrison AK, Mychaliska KP. On target: a case of acute hemorrhagic edema of infancy. Hosp Pediatr 2014;4(2):106-108. doi: 10.1542/hpeds.2013-0054.
23. Hawkrigg S, Johnson A, Flynn J, Thom G, Wright H. Acute haemorrhagic oedema of infancy in a 5-week-old boy referred to the Child Protection Unit. J Paediatr Child Health 2014;50(6):487-489. doi: 10.1111/jpc.12453.
24. Maggio MC, Ferraro F, Ragusa SS, Corpora U, Corsello G. Idiopathic Seidlmayer’s purpura: a case report. Case Rep Dermatol 2014;6(2):150–153. doi: 10.1159/000362754.
25. Mohd Sazlly Lim S, Shamsudin N. Acute haemorrhagic oedema of infancy with bullae and koebnerisation. Malays Fam Physician 2014;9(2):55-57.
26. Nacaroğlu HT, Saygaz S, Sandal ÖS, Karkıner CSÜ, Yıldırım HT, Can D. Acute hemorrhagic edema of infancy after vaccination: a case report. J Pediatr Inf 2014;8:40-43. doi: 10.5152/ced.2013.41.
27. Orden Rueda C, Clavero Montañés N, Berdún Cheliz E, Calvo Ferrer C, Visiedo Fenollar A, Sánchez Gimeno J. Edema agudo hemorrágico. Bol Pediatr Arag Rioj Sor 2014;44(3):64-66.
28. Risikesan J, Koppelhus U, Steiniche T, Deleuran M, Herlin T. Methylprednisolone therapy in acute hemorrhagic edema of infancy. Case Rep Dermatol Med 2014;2014:853038. doi: 10.1155/2014/853038.
29. Robl R, Robl M, Marinoni LP, Abagge KT, Carvalho VO. Target-shaped edematous purple lesions: is it child abuse? Arch Dis Child 2014;99(1):44-45. doi: 10.1136/archdischild-2013-304874.
30. Sorensen EP, Matiz C, Friedlander SF. An 8-month-old boy with purpuric skin lesions. Acute hemorrhagic edema of infancy. Pediatr Ann 2014;43(1):e4-e8. doi: 10.3928/00904481-20131223-09.
31. Argyri I, Korona A, Mougkou K, Vougiouka O, Tsolia M, Spyridis N. Photo quiz. An infant with purpuric rash and edema. Clin Infect Dis 2015;61(10):1553,1624-1625. doi: 10.1093/cid/civ546.
32. Binamer Y. Acute hemorrhagic edema of infancy after MMR vaccine. Ann Saudi Med 2015;35(3):254-256. doi: 10.5144/0256-4947.2015.254.
33. Breda L, Franchini S, Marzetti V, Chiarelli F. Escherichia coli urinary infection as a cause of acute hemorrhagic edema in infancy. Pediatr Dermatol 2015;32(6):e309-e311. doi: 10.1111/pde.12690.
34. Checa Rodríguez R, Carabaño Aguado I, Álvarez Fernández B. Edema agudo hemorrágico en un neonato. Pediatr Aten Primaria 2015;17(66):151-153.
35. Cunha DF, Darcie AL, Benevides GN, Ferronato AE, Hein N, Lo DS, Yoshioka CR, Hirose M, Cardoso DM, Gilio AE. Acute hemorrhagic edema of infancy: an unusual diagnosis for the general pediatrician. Autops Case Rep 2015;5(3):37-41. doi: 10.4322/acr.2015.020.
36. Dhillon M, Dhariwal DK. Acute haemorrhagic oedema of infancy (AHOI): a case report. J Maxillofac Oral Surg 2015;14(Suppl 1):173-175. doi: 10.1007/s12663-012-0405-3.
37. Fraga BSD, Barbosa NM, Miranda BPC, Leite MAR. Edema hemorrágico agudo da infância: um relato de caso. Rev Pediat SOPERJ 2015;15(supl 1)(2):24.
38. Gül N, Çelik T, Kara F, Dilen F, Temiz F, Çelik Ü. Acute infantile hemorrhagic edema: a case and literature review. J Pediatr Inf 2015;9:175-177. doi: 10.5152/ced.2015.1668.
39. Kars V, Yilmaz A, Celepkolu T, Aslanhan H, Arslan N, Demir V. Acute infantile hemorrhagic edema mimicking Henoch-Schönlein purpura. Med Sci Discov 2015;2(2):193-194. doi: 10.17546/msd.17911.
40. Kurtipek GS, Akyürek FT, Ataseven A, Kocaoglu Ç, Küçükosmanoğlu İ. Infantile acute hemorrhagic edema: four attacks in two years. Abant Med J 2015;4(3):269-271. doi: 10.5505/abantmedj.2015.60352.
41. Magro CM. Acute hemorrhagic edema of infancy. Dermatopathologist 2015;23(6):4.
42. Monteiro Lacerda AC, Silva SA, Simão Rafael M, Madeira Correia SA, Barradas Batista C, Ferreira Castanhinha SI. Edema hemorrágico agudo da infância: uma vasculite com bom prognóstico. Sci Med 2015;25(3):21381. doi: 10.15448/1980-6108.2015.3.21381.
43. Nair BT, Krishna SR, Karmani S. Leukocytoclastic vasculitis following DPT vaccination. Trop J Med Res 2015;18:42-44. doi: 10.4103/1119-0388.152695.
44. Oliveira JA, Lopes L, Fraga A, Soares-de-Almeida L, Águas B, Siborro-Azevedo A. Acute hemorrhagic edema of infancy: a rare cause of purpuric exanthema. J Pediatr 2015;166(2):498-98.e1. doi: 10.1016/j.jpeds.2014.09.048.
45. Ostini A, Ramelli GP, Mainetti C, Bianchetti MG, Ferrarini A. Recurrent Finkelstein-Seidlmayer disease in four first-degree relatives. Acta Derm Venereol 2015;95(5):622-623. doi: 10.2340/00015555-2017.
46. Pittet LF, Siebert JN, Lacroix LE. Striking but benign: acute haemorrhagic oedema of infancy. Lancet 2015:S0140-6736(15)00318-9. doi: 10.1016/S0140-6736(15)00318-9.
47. Roy KP, Madke B, Kar S, Yadav N. Acute hemorrhagic edema of infancy. Indian J Dermatol 2015:60(6):624-625. doi: 10.4103/0019-5154.169150.
48. Sarkar S, De A. Urticaria multiforme. Indian Pediatr 2015;52:633. doi: 10.1007/s13312-015-0690-2.
49. Açıkgöz M, Güzel A. Acute hemorrhagic edema of infancy: a case report. J Pediatr Emerg Intensive Care Med 2016;3:46-48. doi: 10.4274/cayd.64936.
50. Ameta G. Acute hemorrhagic edema: rare variety of leukocytoclastic vasculitis. Indian J Paediatr Dermatol 2016;17(4):280-282. doi: 10.4103/2319-7250.184330.
51. Butterfield R, Patel B. Visual diagnosis: infant with ecchymoses. Pediatr Rev 2016;37(10):e41–e44. doi: 10.1542/pir.2015-0119.
52. Caixeta MF, Lima JS, Zandonaide AGB. Acute hemorrhagic edema of childhood: case report and comparison with meningococcemia. Resid Pediátr 2016;6(2):98-102. doi: 10.18554/refacs.v5i1.1918.
53. Efstathiou E, Papamichalopoulou S, Georgiou G, Hadjipanayis A. Rapid onset of purpuric rash in an otherwise healthy 6-month-old infant. Clin Pediatr 2016;56(14):1377-1380. doi: 10.1177/0009922816674524.
54. Homme JL, Block JM. Acute hemorrhagic edema of infancy and common mimics. Am J Emerg Med 2016;34(5):936.e3-6. doi: 10.1016/j.ajem.2015.09.030.
55. Karaman K, Akbayram S, Garipardiç M, Öner AF. Acute infantile hemorrhagic edema: report of two cases. Turk J Pediatr Dis 2016;3:216-218. doi: 10.12956/tjpd.2016.196.
56. Kretschmer AM, Krause B, Buchauer F, Seeliger S. Akutes hämorrhagisches Ödem des Kleinkindes. Monatsschr Kinderheilkd 2016;164:598-602. doi: 10.1007/s00112-015-0015-5.
57. Mreish S, Al-Tatari H. Hemorrhagic lacrimation and epistaxis in acute hemorrhagic edema of infancy. Case Rep Pediatr 2016;2016:9762185. doi: 10.1155/2016/9762185.
58. Neves C, Brito N, Santos D, Mota L. Purpuric lesions: what diagnosis? BMJ Case Rep 2016;2016:bcr2015210154. doi: 10.1136/bcr-2015-210154.
59. Özdemir Ö, Avcı O, Akın İM, Büyükkayhan D. Prognostic importance of D-dimer level in acute infantile hemorrhagic edema. J Curr Pediatr 2016;14:142-146. doi:10.4274/jcp.69775.
60. Pinto PA, Aguiar C, Dinis MJ, Ramos S. Edema agudo hemorrágico do lactente – a (re) conhecer. Birth Growth Med J 2016;25(4):251-254.
61. Serra E Moura Garcia C, Sokolova A, Torre ML, Amaro C. Acute hemorrhagic edema of infancy. Eur Ann Allergy Clin Immunol 2016;48(1):22-26.
62. Al Murayshid AI. Acute haemorrhagic oedema with extensive truck involvement and rhinovirus infection association. J Dermatol Dermatol Surg 2017;21:27–30. doi: 10.1016/j.jdds.2016.08.004.
63. Alvarado Socarras J, Fernandez Velosa ZA. Edema agudo hemorrágico de la infancia. Lesiones alarmantes de un cuadro benigno. Reporte de caso. Arch Argent Pediatr 2017;115(6):e432-e435. doi: 10.5546/aap.2017.e432.
64. Behera B, Singh N, Thappa DM, Rajesh NG. Acute hemorrhagic edema of infancy: panicked parents and playful child. Indian J Paediatr Dermatol 2017;18(1):67-69. doi: 10.4103/2319-7250.184327.
65. Chavez-Alvarez S, Barbosa-Moreno L, Ocampo-Garza J, Ocampo-Candiani J. Acute hemorrhagic edema of infancy (Finkelstein's disease): favorable outcome with systemic steroids in a female patient. An Bras Dermatol 2017;92(1):150-152. doi: 10.1590/abd1806-4841.20175846.
66. Chesser H, Chambliss JM, Zwemer E. Acute hemorrhagic edema of infancy after coronavirus infection with recurrent rash. Case Rep Pediatr 2017;2017:5637503. doi: 10.1155/2017/5637503.
67. Consuegra-Solano J, Agualimpia-Palacios LC, Cadavid-Zapata KL, Kury-Palacios SY, Sánchez IP. Edema agudo hemorrágico de la infancia. CES Med 2017;31(2):192-198. doi: 10.21615/cesmedicina.31.2.8.
68. Debray A, Ollier V, Coutard A Arditty F, Bekkar S, Bodemer C, Leruez-Ville M, Mirand A, Lesage F, Foucaud P. Oedème aigu hémorragique du nourrisson associé à une infection à Coxsackie virus. Arch Pédiatr 2017;24(12):1262-1266. doi: 10.1016/j.arcped.2017.09.013.
69. Elmahi H, Elloudi S, Baybay H, Gallouj S, Mernissi FZ. Acute hemorrhagic edema of infancy: a new observation. Int J Pediat Health Care Adv 2017;4(2):29-30. doi: 10.19070/2572-7354-170009.
70. Neri I, Labriola F, Virdi A, Patrizi A. An impressive, sudden, and purpuric eruption. J Pediatr 2017;191:278‐278. doi: 10.1016/j.jpeds.2017.08.059
71. Parker L, Shahar-Nissan K, Ashkenazi-Hoffnung L, Harel L, Amir J, Trivizki O, Bilavsky E. Acute hemorrhagic edema of infancy: the experience of a large tertiary pediatric center in Israel. World J Pediatr 2017;13(4):341-345. doi: 10.1007/s12519-017-0032-7.
72. Temel EÜ, Öz NA, Temizkan RC, Hıdımoğlu B, Kocabay K. Bad-looking, good-natured disease: acute hemorrhagic edema of infancy. J Curr Pediatr 2017;15:51-54. doi:10.4274/jcp.29484.
73. Van der Heggen T, Dhont E, Schelstraete P, Colpaert J. Acute hemorrhagic edema of infancy: a dramatic presentation with a benign course. Belg Assoc Pediatr 2017;19(1):49.
74. Agarwal N, Taneja S, Bihari S, Verma A. Acute hemorrhagic edema in a nursing infant - An unusual diagnosis. Indian J Child Health 2018;5(4):310-311.
75. Backhouse L, Furnell D, Li Kam Wa I. Unexplained bruising: a developing story. BMJ Case Rep 2018;2018:bcr2017222793. doi: 10.1136/bcr-2017-222793.
76. Bhandari B, Singh R, Kumar M, Saun A. Acute hemorrhagic edema of infancy. Indian J Pediatr 2018;85(3):245-246. doi: 10.1007/s12098-017-2463-5.
77. Bülbül L, Hatipoğlu N, Özkul Sağlam N, Hasbal Akkuş C, Hatipoğlu S. Acute hemorrhagic edema of infancy: a two-case report. Med Bull Sisli Etfal Hosp 2018;52(3):220–223. doi: 10.5350/SEMB.20170419063548.

1. [Ceci M](https://www.ncbi.nlm.nih.gov/pubmed/?term=Ceci%20M%5BAuthor%5D&cauthor=true&cauthor_uid=27228147), [Conrieri M](https://www.ncbi.nlm.nih.gov/pubmed/?term=Conrieri%20M%5BAuthor%5D&cauthor=true&cauthor_uid=27228147), [Raffaldi I](https://www.ncbi.nlm.nih.gov/pubmed/?term=Raffaldi%20I%5BAuthor%5D&cauthor=true&cauthor_uid=27228147), [Pagliardini V](https://www.ncbi.nlm.nih.gov/pubmed/?term=Pagliardini%20V%5BAuthor%5D&cauthor=true&cauthor_uid=27228147), [Urbino AF](https://www.ncbi.nlm.nih.gov/pubmed/?term=Urbino%20AF%5BAuthor%5D&cauthor=true&cauthor_uid=27228147). Acute hemorrhagic edema of infancy: still a challenge for the pediatrician. Pediatr Emerg Care 2018;34(2):e28-e29. doi: 10.1097/PEC.0000000000000749.
2. Chiriac A, Podoleanu C, Naznean A, Stolnicu S. Acute hemorrhagic edema of infancy: a benign condition with impressive clinical picture. Pediatr Neonatol 2018;59(5):538-539. doi: 10.1016/j.pedneo.2018.07.010.
3. Dayananda P, Barrett S, Nair S. A case of human Metapneumovirus associated with acute haemorrhagic oedema of infancy. Clin Med Rev Case Rep 2018;5(4):210. doi.org/10.23937/2378-3656/1410210.
4. Drerup C, Hake L, Oji V, Sunderkötter C. Recurrent acute hemorrhagic edema of infancy (AHEI) during puberty. J Dtsch Dermatol Ges 2018;16(12):1496-1498. doi: 10.1111/ddg.13699.
5. Haeusler IL, Mohan R. Acute haemorrhagic oedema of infancy: a benign cause of a formidable rash. BMJ Case Rep 2018;2018: bcr-2017-223368. doi: 10.1136/bcr-2017-223368.
6. Landau DC, Bosio Bonet M, Salduna MD, Kahn A, Kurpis M, Ruiz Lascano A. Edema hemorrágico agudo del lactante. Arch Argent Pediatr 2018;116(2):166-168.
7. Manzano G, Shantharam R, Webb E, Finelt N, Hengel K. Hemolacria, hematochezia, and hematuria in an 11-month-old boy. Pediatr Rev 2018;39(8):418-420. doi: 10.1542/pir.2016-0155.
8. Rohr BR, Manalo IF, Mowad C. Acute hemorrhagic edema of infancy: guide to prevent misdiagnosis. Cutis 2018;102(5):359-362.
9. Speirs L, McVea S, Little R, Bourke T. What is that rash? Arch Dis Child Educ Pract Ed 2018;103(1):25-26. doi: 10.1136/archdischild-2016-311782.
10. Alharbi MS. Role of systemic steroids in acute hemorrhagic edema of infancy: report of two cases. Int J Adv Med 2019;6(2):537-541. doi: 10.18203/2349-3933.ijam20191172.
11. Bekdaş M, İldeş EN. Acute infantile hemorrhagic edema following Measles Mumps Rubella vaccine: a case report. Abant Med J 2019;8(2):87-89. doi:10.5505/abantmedj.2019.09821.
12. Benli S, Tekin M. A case of acute infantile hemorrhagic edema. Ege J Med 2019;58(1):84-85.
13. Bhattarai D, Guleria S, Suri D. Acute haemorrhagic oedema of infancy: alarming but clinically benign vasculitis. J Paediatr Child Health 2019;55(9):1150-1151. doi: 10.1111/jpc.14545.
14. Carboni E, Scavone M, Stefanelli E, Talarico V, Zampogna S, Galati MC, Raiola G. Case report: acute hemorrhagic edema of infancy (Seidlmayer purpura) - a dramatic presentation for a benign disease. F1000Res 2019;8:1771. doi: 10.12688/f1000research.20645.1.
15. Garcia-Muro C, Esteban-Zubero E. Acute hemorrhagic edema of infant: a case report. Mathews J Pediatr 2019;4(1):15.
16. Kılıçaslan Ö, Yıldız R, Engin MMN, Büyük N, Temizkan RC, Özlü E, Kocabay K. Acute infantile hemorrhagic edema clinic: two case reports. J Acad Res Med 2019;9(2):111-114.
17. Miconi F, Cassiani L, Savarese E, Celi F, Papini M, Principi N, Esposito S. Targetoid skin lesions in a child: acute hemorrhagic oedema of infancy and its differential diagnosis. Int J Environ Res Public Health 2019;16(5):e823. doi: 10.3390/ijerph16050823.
18. Rinoldi PO, Milani GP, Bianchetti MG, Ferrarini A, Ramelli GP, Lava SAG. Acute hemorrhagic edema of young children: open questions and perspectives. Int J Dermatol Skin Care 2019;1:63‐67.
19. Spagnut G, Silecchia V, Valerio E, Cutrone M, Grimalt R. Isolated acute hemorrhagic edema of the eyelids. J Dermatol Res 2019;4(2):172-173. doi:10.17554/j.issn.2413-8223.2019.04.46-3.
20. Thakur P, Negi B, Chandrashekar, Attri P, Basit R, Bhardwaj V, Gupta R. Acute haemorrhagic oedema of infancy. J Med Sci Clin Res 2019;7(8):549-551. doi: 10.18535/jmscr/v7i8.92.
21. Varanaki ME, Ladomenou F, Anatoliotakj M, Vlachaki G. Review and case report of acute hemorrhagic edema of infancy. A benign cause of a striking rash. Eur J Pediat Dermatol 2019;29(3):134-138. doi: [10.26326/2281-9649.29.3.1999](https://doi.org/10.26326/2281-9649.29.3.1999).
22. Yalçın K, Girişgen İ, Polat M, Demirkan Çallı N, Yüksel S. A case report of acute infantile hemorrhagic edema with adenovirus infection. Pam Med J 2019;12(2):333-336. doi: 10.31362/patd.474977.
23. Cucinotta U, Mazza F, Pajno GB, Gallizzi R. Acute haemorrhagic oedema of infancy: a condition that is not always benign. BMJ Case Rep 2020;13:e236059. doi:10.1136/bcr-2020-236059.
24. Edwards MO, El Briri A. Purpuric rash in an infant after chicken pox exposure. Oxf Med Case Reports 2020(1):omz142. doi: 10.1093/omcr/omz142.
25. Gao PR, Yen H, Chen WT. Acute hemorrhagic edema of infancy. CMAJ 2020 26;192:E1309. doi: 10.1503/cmaj.200418.
26. Haddad GR, Protásio IR, Fioretto JR, Cardoso LF, Romero FR, Martin JG. Acute hemorrhagic edema of infancy: a case series of three cases. Indian J Case Reports 2020;6(12):676-678. doi: 10.1590/s0365-05962011000600019.
27. Relhan V, Sandhu J, Garg VK, Khurana N. A rare vasculitis with a dramatic presentation: acute hemorrhagic edema of infancy. Indian J Paediatr Dermatol 2020;21:215-217. doi: 10.4103/ijpd.IJPD_84_18.
28. Riola Blanco AM, López Pacios D, Serrano Crespo N. Edema agudo hemorrágico del lactante. Rev Pediatr Aten Primaria 2020;21(84):397-399.
29. Salam A, Zaman S, Banerjee P. A rapidly evolving rash in a well child. Paediatr Child Health 2020;25(3):129-130. doi: 10.1093/pch/pxz009.
30. Shahbaznejad L, Navaeifar MR, Abbaskhanian A, Hosseinzadeh F, Rahimzadeh G, Rezai MS. Clinical characteristics of 10 children with a pediatric inflammatory multisystem syndrome associated with COVID-19 in Iran. BMC Pediatr 2020;148:e196. doi: [10.1017/S095026882000196X](https://dx.doi.org/10.1017%2FS095026882000196X).
31. Wadhwa A, Patra AK, Dharman BK. A case of acute hemorrhagic edema of infancy. Our Dermatol Online 2020;11(2):146-148. doi: 10.7241/ourd.20202.7.
32. Contorno S, Cozzi G, Berti I, Barbi E, Taddio A. Young child with painful edema and purpura: a case report. Ital J Pediatr 2021;47(1):61. doi: 10.1186/s13052-021-01001-y.
33. Dewan P, Singal A. Childhood Rashes: A Pediatrician's Dilemma. Indian Pediatr. 2021;58(1):99. doi: 10.1007/s13312-021-2122-9.
34. Hancock R, Rothman I, Mannix MK, Islam S. Infant with a rapidly progressing rash. BMJ Case Rep 2021;14:e239353. doi: 10.1136/bcr-2020-239353.
35. Heck E, Johnson N, Ramien M. Autoinflammatory disease mimicking acute hemorrhagic edema of infancy. Pediatr Dermatol 2021;38(1):223-225. doi: 10.1111/pde.14402.
36. Hoskins B, Keeven N, Dang M, Keller E, Nagpal R. A child with COVID-19 and immunoglobulin a vasculitis. Pediatr Ann 2021;50(1):e44-e48. doi: 10.3928/19382359-20201211-01.
37. Janssen NE, Keukens L. Indrukwekkend huidbeeld bij een gezond kind. Huisarts Wet 2021;64:1-3. doi:10.1007/s12445-020-0994-x.
38. Medovic R, Medovic M, Igrutinovic Z, Vlahovic A, Kostic G, Ristic G, Vuletic B, Markovic S. Acute hemorrhagic edema of infancy - is it really a mild, benign disease? Turk J Pediatr 2021;63(1):141-148. doi:10.24953/turkjped.2021.01.017.
39. O'Connor C, Bux D, O'Connell M. Acute haemorrhagic oedema of infancy: first report of a rare small vessel vasculitis in the neonatal period. Arch Dis Child. 2021;106(6):582. doi: 10.1136/archdischild-2020-319739.
40. Saliba E, Sayad A, Alameddine L, El-Haddad K, Tannous Z. Mycoplasma pneumonia and atypical acute hemorrhagic edema of infancy. Am J Emerg Med 2021;41:266.e3-266.e5. doi: 10.1016/j.ajem.2020.08.067.
41. Saraiva BM, Lobato MB, Santos E, Garcia AM. Acute haemorrhagic oedema of infancy as a manifestation of COVID-19. BMJ Case Rep 2021;14:e241111. doi:10.1136/bcr-2020-241111.
42. Sneller H, Vega C, Zemel L, Chicaiza HP. Acute hemorrhagic edema of infancy with associated hemorrhagic lacrimation. [Pediatr Emerg Care](https://www.ncbi.nlm.nih.gov/pubmed/30113438) 2021;37(2):e70-e72. doi: 10.1097/PEC.0000000000001542.
